# Modulating Treatment Outcomes of Patients with Solid Tumors in Immunotherapy Trials: A Drug Interaction Analysis from a Phase I Unit

**DOI:** 10.1158/2767-9764.CRC-25-0033

**Published:** 2025-09-15

**Authors:** Camila Braganca Xavier, Clark R. Andersen, JoAnn Lim, Julian H. Slade, Stacie A. Bean, Lei Kang, Hung Le, Apostolia M. Tsimberidou, Aung Naing, David S. Hong, Ecaterina E. Dumbrava, Jordi Rodon Ahnert, Paula R. Pohlmann, Sarina A. Piha-Paul, Stephane Champiat, Timothy A. Yap, Tin-Yun Tang, Funda Meric-Bernstam, Siqing Fu

**Affiliations:** 1 Department of Investigational Cancer Therapeutics, The University of Texas MD Anderson Cancer Center, Houston, Texas.; 2 Department of Biostatistics, The University of Texas MD Anderson Cancer Center, Houston, Texas.

## Abstract

**Purpose:**

Concurrent use of medications can modulate the effectiveness of immunotherapy. Although this interaction is well documented for immune checkpoint inhibitors, whether this occurs with new experimental compounds has not been evaluated.

**Patients and Methods:**

A computerized data extraction tool was used to collect clinical data and identify the prescription of a predefined set of medications within 30 days of immunotherapy infusion in the Department of Investigational Cancer Therapeutics at the University of Texas MD Anderson Cancer Center. The primary endpoints were median overall survival (OS) and progression-free survival. Tumor responses were assessed using RECIST.

**Results:**

We identified 897 patients. The most prevalent tumor types were colorectal (24.5%), head and neck (10.5%), and pancreatic (9.4%). The immunotherapy administered consisted of monoclonal antibodies and fusion proteins (64.7%), immune modulators (IM; 20.8%), combinations of IMs and antibodies (9.2%), and oncolytic viruses and cancer vaccines (5.3%). The most frequently prescribed drugs were narcotics (70.5%), antiemetics (49.1%), antihistamines (34.6%), antibiotics (31.2%), and proton pump inhibitors (PPI; 28.7%). Patients receiving antihistamines exhibited increased rates of stable disease and partial response (*χ*^2^ 8.48; *P* = 0.014) on the IMs and antibodies combination. The benefit of antihistamines was confirmed in a multivariate analysis of OS [HR, 0.752 (95% CI, 0.603–0.938); *P* = 0.012]. For patients with colorectal cancer, PPI use was associated with shortened survival, with a median OS of 5.2 months with PPI use and 8.6 months without it (*P* < 0.001).

**Conclusions:**

Our findings highlight the need for strategies to guide concurrent medication choices for patients receiving immunotherapy in early-phase trials.

**Significance:**

Concurrent administration of antihistamines correlates with enhanced survival in patients receiving experimental immunotherapy for cancer. Conversely, PPI use diminishes survival in patients with colorectal cancer. These findings highlight how tumor immunogenicity and drug interactions can modulate response and survival outcomes, offering new insights to optimize investigational immunotherapy.

## Introduction

The spectrum of immunotherapy compounds administered to patients with solid tumors has been shifting over the past few years, moving from almost exclusively classical immune checkpoint inhibitors (ICI) targeting cytotoxic T-lymphocyte antigen 4, as well as PD-1 and its ligand, PD-L1 (1), to a broader portfolio of therapies, including newer antibodies, fusion proteins (FP), immune modulators (IM), oncolytic viruses (OV), and cancer vaccines (2). The concurrent use of medications can either compromise or enhance the efficacy of ICIs as demonstrated in populational (3, 4) and *in vitro* (5) studies. However, their effects on the outcomes of newer experimental immunotherapy compounds are unknown. Therefore, the objective of the present retrospective study was to determine whether the concurrent use of a predefined set of medications within 30 days of investigational immunotherapy infusion is associated with survival. This assessment is highly important because patients with cancer are increasingly undergoing treatment in early-phase clinical trials, and protocol development must guarantee optimized safety and the chance of success of investigational therapies (6).

## Materials and Methods

To investigate the effect of concurrent medications on the treatment outcomes in patients enrolled in early-phase clinical trials evaluating immunotherapy in the Department of Investigational Cancer Therapeutics at the University of Texas MD Anderson Cancer Center from June 2014 to June 2024, a computerized data extraction tool was used to collect clinical data and identify the prescription of a predefined set of medications within 30 days of the experimental immunotherapy infusion (Supplementary Table S1). Trials evaluating immunotherapy in combination with chemotherapy or targeted therapy were excluded. Patients enrolled in more than one immunotherapy trial sequentially were also excluded. Tumor types were classified as immunoresponsive if they had standard-of-care FDA-approved immunotherapy. Otherwise, they were classified as immunorefractory. The primary endpoints were the median overall survival (mOS) duration, defined as the time from the first treatment cycle to death or the last follow-up visit, and the median progression-free survival (mPFS) duration, defined as the time from the first treatment cycle to disease progression or death owing to any cause. Tumor responses were categorized using the RECIST (7). The disease control rate (DCR) was defined as the proportion of patients having a complete response, a partial response (PR), or stable disease (SD) as the best response. The Kaplan–Meier method was used to summarize OS and PFS, as well as OS according to whether the patient received treatment with proton pump inhibitors (PPI).

A *χ*^2^ test was performed to evaluate the correlation of medication use with the type of response. Cox univariate proportional hazards models were used to assess the associations of OS and PFS with specific study variables, including age at cycle 1 day 1, body mass index (BMI), sex, race, age of at least 65 years, discrete BMI category, cancer group (immunorefractory vs. immunoresponsive), metastasis count (no more than two vs. two or more), albumin level group (normal vs. low), therapy group (antibodies and FPs or IMs with or without antibodies), best response [progressive disease (PD), SD, or PR], and binary status of pain, diabetes, and nonsteroidal anti-inflammatory drug (NSAID); antihistamine; steroid; PPI; antibiotic; antidepressant; antiemetic; antidiabetic medication; anticoagulant; immunosuppressant; bone-modifying agent; and narcotic use.

A Cox multivariable model for OS was based on exhaustive variable selection over the variables sex, race, age of at least 65 years, discrete BMI category, cancer group, metastasis count (no more than two vs. two or more), albumin level group, therapy group, best response, and binary status of pain, diabetes, and NSAID; antihistamine; steroid; PPI; antibiotic; antidepressant; antiemetic; antidiabetic medication; anticoagulant; immunosuppressant; bone-modifying agent; and narcotic use with the optimal model determined per the combination of variables, which yielded the lowest value for the Akaike information criterion. The resulting optimal model included the variables discrete BMI category, cancer group (immunorefractory vs. immunoresponsive), albumin group (normal vs. low), best response (PD, SD, or PR), and binary statuses of antihistamine, steroid, PPI, antibiotic, immunosuppressant, bone-modifying agent, and narcotic use. A similar approach was used for a multivariable model of PFS; the resulting model included the variables discrete BMI category, cancer group (immunorefractory vs. immunoresponsive), metastasis count of no more than two, albumin group (normal vs. low), and binary statuses of pain, diabetes, and NSAID use. All statistical analyses were performed using R statistical software (version 4.4.1) and JASP computer software (version 0.19.3). The criterion for significance was set at *P* < 0.05.

### Data availability

The data generated in this study are not publicly available owing to information that could compromise patient privacy but are available from the corresponding author upon reasonable request.

## Results

### Patients’ characteristics

We identified a total of 897 patients. We excluded 70 patients from the analysis owing to enrollment in two or more sequential immunotherapy protocols. Data for the remaining 827 patients (49.3% female, 50.7% male) are summarized in Table 1. Most of the patients were White (72.9%), and their median age was 58 years (range, 20–85 years). The most prevalent tumor types were colorectal (24.5%), head and neck (10.5%), and pancreatic (9.4%). The cohort also included patients with rare histologies such as adrenal cancer (0.4%), germ cell tumors (0.6%), cancers of the thymus (0.8%), thyroid cancer (0.1%), and cancer of unknown primary (0.4%). Most patients had two or more metastatic tumor sites (85.7%). Cancer-related pain occurred in 74% of the patients. The immunotherapies administered were monoclonal antibodies and FPs (64.7%), IMs (20.8%), IMs and antibodies (9.2%), and OVs and cancer vaccines (5.3%). The most frequently prescribed drugs from the predefined set of medications were narcotics (70.5%), antiemetics (49.1%), antihistamines (34.6%), antibiotics (31.2%), and PPIs (28.7%).

**Table 1.** Patients’ characteristics (*N* = 827).

Characteristic	*N* (%)
Sex	​
Female	408 (49)
Male	419 (51)
Race/ethnicity	​
American Indian or Alaska Native	3 (0.4)
Asian	66 (8.0)
Black or African American	81 (9.8)
Hispanic or Latino	9 (1.1)
Native Hawaiian or other Pacific Islander	3 (0.4)
Other/unknown	62 (7.5)
White	603 (72.9)
Median age, years (range)	58 (20–85)
Tumor type	​
Adrenal	3 (0.4)
Anal	9 (1.1)
Biliary tract	20 (2.4)
Breast	73 (8.8)
Cervical	14 (1.7)
CNS	4 (0.5)
Colorectal	203 (24.5)
CUP	3 (0.4)
Endometrial	20 (2.4)
Esophagogastric	54 (6.5)
Germ cell	5 (0.6)
Head and neck	87 (10.5)
Liver	4 (0.5)
Lung	46 (5.6)
Melanoma	23 (2.8)
Mesothelioma of peritoneum	5 (0.6)
Mesothelioma of pleura	5 (0.6)
Neuroendocrine	10 (1.2)
Ovarian/fallopian tube	53 (6.4)
Pancreatic	78 (9.4)
Penile	1 (0.1)
Prostate	19 (2.3)
Sarcoma	42 (5.1)
Skin (nonmelanoma)	13 (1.6)
Thymus	7 (0.8)
Thyroid	1 (0.1)
Urachus	2 (0.2)
Urothelial	23 (2.8)
Tumor group	​
Immunorefractory	567 (68.6)
Immunoresponsive	260 (31.4)
Metastasis count	​
≤2	112 (13.5)
>2	709 (85.7)
N/A	6 (0.7)
Albumin level	​
Normal	752 (90.9)
Low	73 (8.8)
N/A	2 (0.2)
BMI category	​
Underweight/normal	310 (37.5)
Overweight	231 (27.9)
Obese	213 (25.8)
N/A	73 (8.8)
Therapy type	​
Antibodies and FPs	535 (64.7)
IM	172 (20.8)
IM + antibody	76 (9.2)
OV	25 (3.0)
OV + antibody	11 (1.3)
Vaccine	6 (0.7)
Vaccine + antibody	2 (0.2)
Cancer-related pain	​
Absent	215 (26.0)
Present	612 (74.0)
Diabetes	​
Absent	605 (73.2)
Present	222 (26.8)
NSAID use	​
Absent	699 (84.5)
Present	128 (15.5)
Antihistamine use	​
Absent	541 (65.4)
Present	286 (34.6)
Steroid use	​
Absent	630 (76.2)
Present	197 (23.8)
PPI use	​
Absent	590 (71.3)
Present	237 (28.7)
Antibiotic use	​
Absent	569 (68.8)
Present	258 (31.2)
Antidepressant use	​
Absent	735 (88.9)
Present	92 (11.1)
Antiemetic use	​
Absent	421 (50.9)
Present	406 (49.1)
Antidiabetic drug use	​
Absent	754 (91.2)
Present	73 (8.8)
Anticoagulant use	​
Absent	620 (75.0)
Present	207 (25.0)
Immunosuppressant use	​
Absent	809 (97.8)
Present	18 (2.2)
Bone-modifying agent use	​
Absent	767 (92.7)
Present	60 (7.3)
Narcotic use	​
Absent	244 (29.5)
Present	583 (70.5)

Abbreviations: CNS, central nervous system; CUP, cancer of unknown primary; N/A, not available.

### Modulating response to treatment with concurrent medications

**Figure 1.**
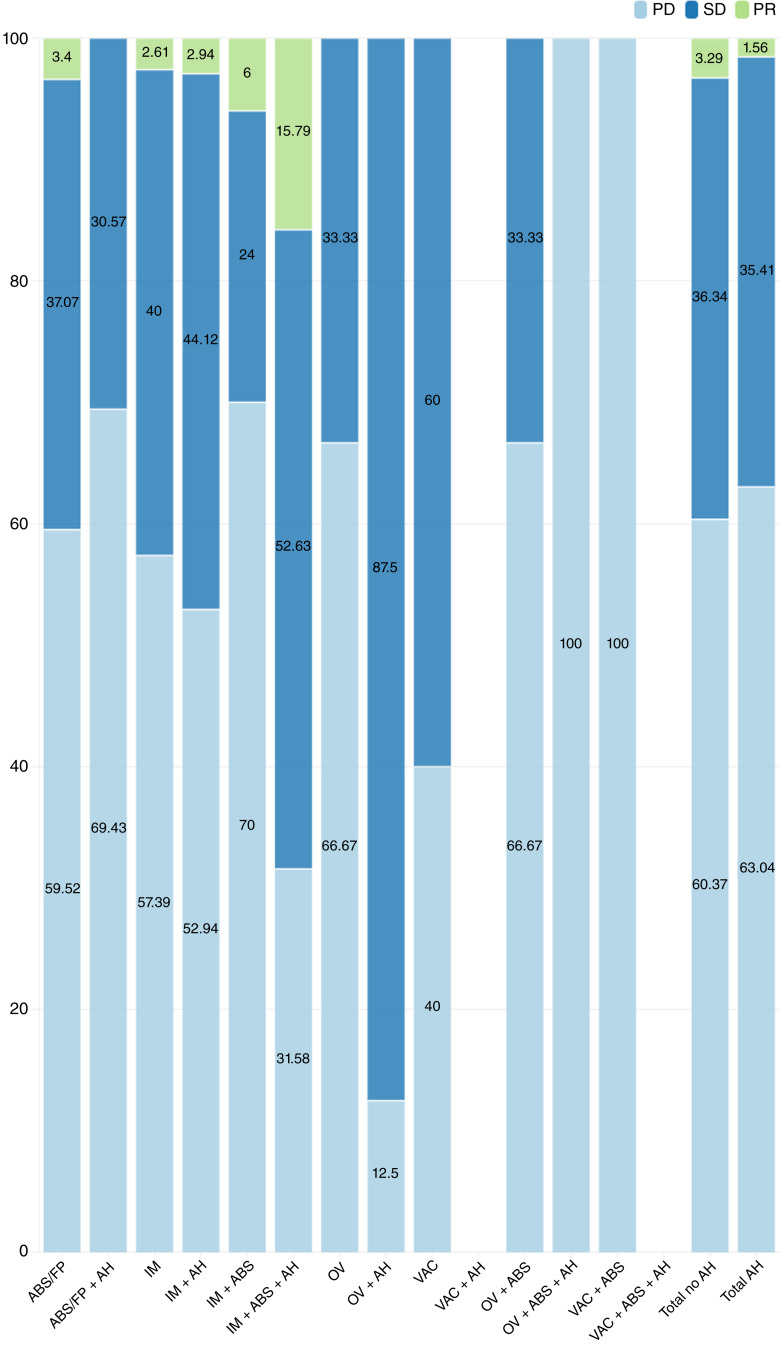
Associations between antihistamine use and tumor responses. ABS, antibodies; AH, antihistamines; VAC, vaccines.

The antitumor response distribution according to treatment type is summarized in Table 2. We observed that 744 patients (90%) with measurable disease had evaluable response data. Of these patients, 456 (61%) had PD as the best response to treatment, whereas 268 (36%) had SD and 20 (3%) had a PR. The overall DCR was 38.7%. When evaluating the different therapy types, the DCR was 36.5% for antibodies and FPs, 43.6% for IMs, 40.6% for IMs and antibodies, and 52.2% for OVs. Among the less frequently prescribed regimens, the DCR ranged from 0% to 60% (*χ*^2^ 19.280; *P* = 0.082). Notably, for the combination of IMs and antibodies, patients who received antihistamines within 30 days of the infusion date exhibited higher rates of SD and PR than did patients who did not have documented use of antihistamines (*χ*^2^ 8.48; *P* = 0.014). We noticed the opposite effect for patients given antibodies and FPs (*χ*^2^ 9.76; *P* = 0.007) as shown in Fig. 1.

**Table 2.** Response data according to therapy type.

Therapy type	Best response, *N* (%)			
	PD	SD	PR	DCR (SD + PR)
Antibodies and FPs (*N* = 487)	309 (63)	168 (34)	10 (2)	178 (37)
IM (*N* = 149)	84 (56)	61 (41)	4 (3)	65 (44)
IM + antibody (*N* = 69)	41 (59)	22 (32)	6 (9)	28 (41)
OV (*N* = 23)	11 (48)	12 (52)	0	12 (52)
OV + antibody (*N* = 9)	7 (78)	2 (22)	0	2 (22)
Vaccine (*N* = 5)	2 (40)	3 (60)	0	3 (60)
Vaccine + antibody (*N* = 2)	2 (100)	0	0	0
Total (*N* = 705)	456 (65)	268 (38)	20 (3)	288 (41)

### Modulation of OS according to concurrent medication

**Figure 2.**
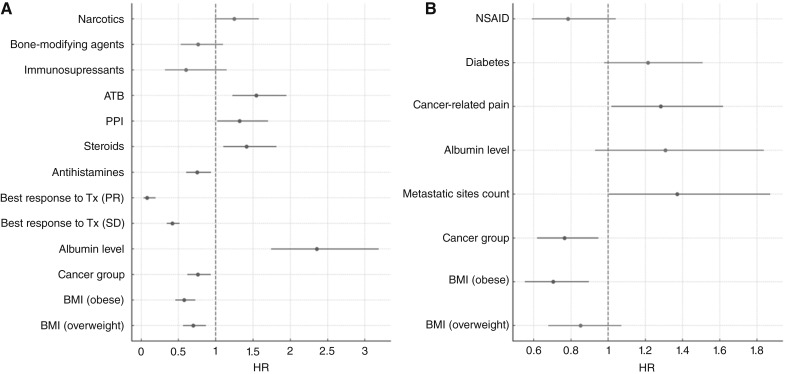
Forest plot for OS (**A**) and PFS (**B**) according to multivariate analysis. ATB, antibiotics.

**Figure 3.**
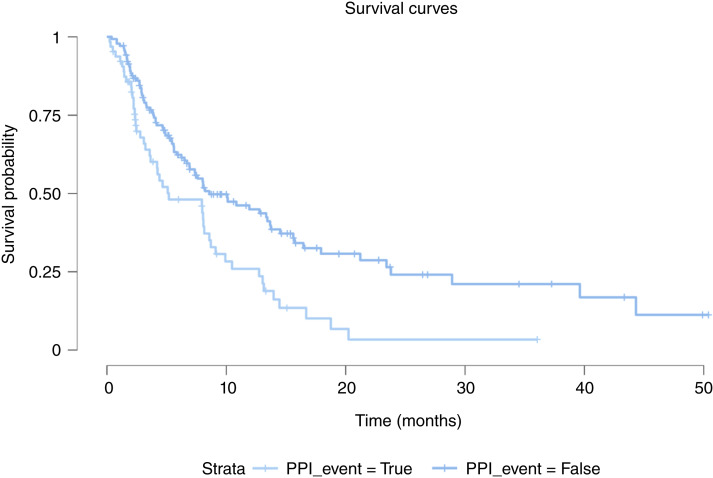
Kaplan–Meier OS curves for patients with colorectal cancer receiving PPIs.

The median time from the first appointment in the MD Anderson Department of Investigational Cancer Therapeutics to death was 9.260 months (range, 0.854–112 months). The mOS duration of the cohort after their initial immunotherapy was 6.140 months (range, 0.164–103 months). At the univariate level, immune-responsive histologies were related to better OS than immunorefractory histologies [HR, 0.738 (95% CI, 0.618–0.883); *P* = 0.0009]. Low albumin level [HR, 2.494 (95% CI, 1.923–3.234); *P* < 0.0001], cancer-related pain [HR, 1.283 (95% CI, 1.060–1.553); *P* = 0.011], low BMI [HR, 0.975 (95% CI, 0.961–0.988); *P* = 0.0002], and the use of steroids [HR, 1.432 (95% CI, 1.191–1.722); *P* = 0.0001], PPIs [HR, 1.738 (95% CI, 1.459–2.069); *P* < 0.0001], antibiotics [HR, 1.699 (95% CI, 1.432–2.017); *P* < 0.0001], antidepressants [HR, 1.308 (95% CI, 1.025–1.670); *P* = 0.031], antiemetics [HR, 1.357 (95% CI, 1.153–1.598); *P* = 0.0002], anticoagulants [HR, 1.552 (95% CI, 1.297–1.858); *P* < 0.0001], and narcotics [HR, 1.394 (95% CI, 1.162–1.673); *P* = 0.0004] were linked with poor OS. Multivariate analysis demonstrated that overweight [HR, 0.697 (95% CI, 0.560–0.869); *P* = 0.003] or obese [HR, 0.575 (95% CI, 0.457–0.725); *P* < 0.0001] status with reference to underweight/normal status, immune-responsive histologies [HR, 0.760 (95% CI, 0.619–0.933); *P* = 0.009], and antihistamine use [HR, 0.752 (95% CI, 0.603–0.938); *P* = 0.012] remained significantly associated with improved survival. Low albumin level [HR, 2.355 (95% CI, 1.741–3.186); *P* < 0.0001], as well as the use of steroids [HR, 1.413 (95% CI, 1.101–1.814); *P* = 0.007], PPIs [HR, 1.318 (95% CI, 1.020–1.703); *P* = 0.035], and antibiotics [HR, 1.542 (95% CI, 1.222–1.947); *P* = 0.0003] remained significantly associated with reduced OS duration. A forest plot of the OS multivariate analysis results is presented in Fig. 2A. In particular, for patients with colorectal cancer, the use of PPIs was associated with a substantial reduction in survival duration, with an mOS time of 5.2 months with PPI use but 8.6 months without it (*P* < 0.001; Fig. 3).

### Modulation of PFS according to concurrent medication

The mPFS duration was 2.07 months (range, 0–37.80 months). In the univariate analysis, an age of at least 65 years [HR, 0.812 (95% CI, 0.665–0.993); *P* = 0.043] and obesity [HR, 0.720 (95% CI, 0.567–0.914); *P* = 0.013] were related to better PFS than were an age younger than 65 years and normal weight, respectively. Also, the presence of cancer-related pain was associated with worse PFS [HR, 1.341 (95% CI, 1.080–1.665); *P* = 0.008]. With respect to the results of the multivariate analysis adjusted according to BMI category, cancer type, metastasis count, albumin level, presence of cancer-related pain, diagnosis of diabetes, and use of NSAIDs, obesity [HR, 0.704 (95% CI, 0.553–0.896); *P* = 0.008] and immunoresponsive histologies [HR, 0.766 (95% CI, 0.619–0.948); *P* = 0.014] were related to better PFS, whereas having more than two metastatic tumor sites [HR, 1.371 (95% CI, 1.004–1.871); *P* = 0.047] and cancer-related pain [HR, 1.283 (95% CI, 1.017–1.618); *P* = 0.036] were related to worse PFS. A forest plot of the PFS multivariate analysis results is presented in Fig. 2B. A summary of the variables related to mOS and mPFS is presented in Table 3.

**Table 3.** Summary of variables related to survival.

Variable comparison	Analysis level	HR (95% CI)	*P* value
OS	​	​	​
Age (absolute)	Uni	1.003 (0.996–1.010)	0.3600
Age group (≥65 vs. <65 years)	Uni	1.045 (0.879–1.242)	0.6200
BMI (absolute)	Uni	0.975 (0.961–0.988)	0.0002
BMI (overweight vs. underweight/normal)	Uni	0.736 (0.600–0.902)	0.0060
​	Multi	0.697 (0.560–0.869)	0.0030
BMI (obese vs. underweight/normal)	Uni	0.630 (0.511–0.778)	<0.0001
​	Multi	0.575 (0.457–0.725)	<0.0001
Sex (male vs. female)	Uni	1.034 (0.878–1.217)	0.6900
Race (Black/African American vs. White)	Uni	1.130 (0.859–1.487)	0.6900
Race (Asian vs. White)	Uni	0.784 (0.555–1.107)	0.3700
Race (other/unknown vs. White)	Uni	1.090 (0.828–1.435)	0.8400
Cancer type (IRESP vs. IREFRA)	Uni	0.738 (0.618–0.883)	0.0009
​	Multi	0.760 (0.619–0.933)	0.0090
Metastasis site count (>2 vs. ≤2)	Uni	1.241 (0.977–1.577)	0.0800
Albumin level (low vs. normal)	Uni	2.494 (1.923–3.234)	<0.0001
​	Multi	2.355 (1.741–3.186)	<0.0001
Therapy type (IM vs. Abs/FP)	Uni	0.874 (0.708–1.079)	0.3500
Therapy type (IM + Abs vs. Abs/FP)	Uni	0.872 (0.649–1.171)	0.5600
Best response to treatment (SD vs. PD)	Uni	0.432 (0.357–0.523)	<0.0001
​	Multi	0.419 (0.342–0.514)	<0.0001
Best response to treatment (PR vs. PD)	Uni	0.088 (0.036–0.216)	<0.0001
​	Multi	0.078 (0.032–0.193)	<0.0001
Cancer-related pain (present vs. absent)	Uni	1.283 (1.060–1.553)	0.0110
Diabetes	Uni	1.092 (0.911–1.308)	0.3400
NSAIDs	Uni	1.030 (0.820–1.295)	0.8000
Antihistamines	Uni	1.125 (0.950–1.332)	0.1700
​	Multi	0.752 (0.603–0.938)	0.0120
Steroids	Uni	1.432 (1.191–1.722)	0.0001
​	Multi	1.413 (1.101–1.814)	0.0070
PPI	Uni	1.738 (1.459–2.069)	<0.0001
​	Multi	1.318 (1.020–1.703)	0.0350
ATB	Uni	1.699 (1.432–2.017)	<0.0001
​	Multi	1.542 (1.222–1.947)	0.0003
Antidepressants	Uni	1.308 (1.025–1.670)	0.0310
Antiemetics	Uni	1.357 (1.153–1.598)	0.0002
Antidiabetics	Uni	1.159 (0.873–1.538)	0.3100
Anticoagulants	Uni	1.552 (1.297–1.858)	<0.0001
Immunosuppressants	Uni	0.938 (0.541–1.626)	0.8200
​	Multi	0.602 (0.317–1.146)	0.1200
Bone-modifying agents	Uni	0.866 (0.630–1.190)	0.3800
​	Multi	0.763 (0.530–1.098)	0.1500
Narcotics	Uni	1.394 (1.162–1.673)	0.0004
​	Multi	1.249 (0.988–1.579)	0.0620
PFS	​	​	​
Age (absolute)	Uni	0.992 (0.984–1.000)	0.0400
Age group (≥65 vs. <65 years)	Uni	0.812 (0.665–0.993)	0.0430
BMI (absolute)	Uni	0.982 (0.967–0.997)	0.0210
BMI (overweight vs. underweight/normal)	Uni	0.867 (0.693–1.086)	0.3600
​	Multi	0.852 (0.678–1.071)	0.2900
BMI (obese vs. underweight/normal)	Uni	0.720 (0.567–0.914)	0.0130
​	Multi	0.704 (0.553–0.896)	0.0080
Sex (male vs. female)	Uni	0.938 (0.780–1.128)	0.4900
Race (Black/African American vs. White)	Uni	1.090 (0.801–1.484)	0.8700
Race (Asian vs. White)	Uni	0.898 (0.628–1.284)	0.8500
Race (other/unknown vs. White)	Uni	1.043 (0.756–1.438)	0.9700
Cancer type (IRESP vs. IREFRA)	Uni	0.756 (0.617–0.926)	0.0070
​	Multi	0.766 (0.619–0.948)	0.0140
Metastasis site count (>2 vs. ≤2)	Uni	1.460 (1.094–1.950)	0.0100
​	Multi	1.371 (1.004–1.871)	0.0470
Albumin level (low vs. normal)	Uni	1.325 (0.950–1.848)	0.1000
​	Multi	1.307 (0.930–1.837)	0.1200
Therapy type (IM vs. Abs/FP)	Uni	0.855 (0.671–1.089)	0.3500
Therapy type (IM + Abs vs. Abs/FP)	Uni	0.844 (0.605–1.178)	0.5100
Best response to treatment (SD vs. PD)^a^	Uni	0 (0–Inf)	1.0000
Best response to treatment (PR vs. PD)^a^	Uni	0 (0–Inf)	1.0000
Cancer-related pain (present vs. absent)	Uni	1.341 (1.080–1.665)	0.0080
​	Multi	1.283 (1.017–1.618)	0.0360
Diabetes	Uni	1.213 (0.988–1.489)	0.0650
​	Multi	1.214 (0.978–1.508)	0.0800
NSAIDs	Uni	0.804 (0.612–1.057)	0.1200
​	Multi	0.784 (0.590–1.041)	0.0900
Antihistamines	Uni	0.986 (0.813–1.195)	0.8800
Steroids	Uni	1.039 (0.838–1.287)	0.7300
PPI	Uni	1.142 (0.931–1.402)	0.2000
ATB	Uni	0.984 (0.804–1.204)	0.8700
Antidepressants	Uni	0.924 (0.683–1.250)	0.6100
Antiemetics	Uni	1.027 (0.854–1.235)	0.7800
Antidiabetics	Uni	1.283 (0.934–1.763)	0.1200
Anticoagulants	Uni	0.991 (0.799–1.228)	0.9300
Immunosuppressants	Uni	1.136 (0.607–2.127)	0.6900
Bone-modifying agents	Uni	0.980 (0.694–1.384)	0.9100
Narcotics	Uni	1.101 (0.896–1.352)	0.3600

Abbreviations: Abs, antibodies; ATB, antibiotic; Inf, infinite; IREFRA, immune-refractory; IRESP, immune-responsive; Multi, multivariate; Uni, univariate.

a Because of the small number of observations in the PR category, the analysis results are not conclusive owing to convergence issues.

## Discussion

In this work, we highlighted the effect of concurrent treatment with drugs on response and survival outcomes in patients with cancer receiving experimental immunotherapy compounds in a phase I clinical setting. In our multivariate analysis, high BMI was associated with improved survival, favoring patients who are overweight and obese over those who are underweight and of normal weight. Additionally, tumors with immune-responsive histologies were linked to better OS outcomes than tumors with immunorefractory histologies, whereas a low albumin level was related to worse OS than a normal level. With respect to specific concurrent therapies, patients receiving antihistamines within 30 days of immunotherapy had better response rates (in selected cases) and survival than patients not receiving antihistamines. Conversely, steroids, PPIs, and antibiotics were associated with poorer survival outcomes.

In recent years, the obesity paradox in cancer immunotherapy has been increasingly studied. Recent research endeavors have demonstrated that obesity-associated metabolic and inflammatory signals drive tumor-associated macrophages to upregulate PD-1 expression, generating a tumor-associated macrophage–specific feedback loop that compromises tumor immune surveillance. This may help explain why obesity correlates with an increased cancer risk yet also confers an improved response to anti–PD-1 immunotherapy (8). However, the mechanisms underlying the interactions between other categories of immunotherapy and obesity remain unclear.

With respect to antihistamine use, we found an association with improved response and survival outcomes in patients receiving immunotherapy. This association was most obvious in patients receiving IMs and antibodies. In the clinical setting, another retrospective cohort study at our institution demonstrated that patients with melanoma receiving H1-specific antihistamines while also receiving anti–PD-1/PD-L1 ICIs had a markedly lower mortality rate than age-, sex-, and stage-matched controls not receiving these agents. Similarly, among patients with non–small cell lung cancer receiving anti–PD-1/PD-L1 ICIs, those who took H1 antihistamines experienced a markedly lower mortality rate compared with those not receiving this class of drugs. Although patients with breast or colorectal cancer also exhibited trends toward reduced mortality rates when taking H1 antihistamines, these differences were not significant, likely because of the smaller patient populations in these groups. A preclinical exploration demonstrated that the histamine H1 receptor is upregulated in the tumor microenvironment and correlates with T-cell dysfunction. Experimentally, in colorectal cancer and melanoma cell models, inhibition of the histamine H1 receptor in macrophages can restore T-cell antitumor immunity, demonstrating that patients with cancer receiving H1 antihistamines concurrently with ICIs experienced improved OS (5). Although the correlation among ICIs, antihistamines, and survival has been documented before, whether IMs could interfere with this association remains unclear.

Evaluating size-based endpoints in early-phase clinical trials can be challenging, as these patients are often heavily pretreated, and lower response rates are commonly observed in this setting. Although the overall response rate (ORR) in phase I clinical trials nearly doubled between 2000 and 2019, response rates elicited by novel agents administered as monotherapy can remain low. Analysis from a pooled cohort from the Cancer Therapy Evaluation Program of the NCI-sponsored investigator-initiated phase I trials for solid tumors, comprising 465 studies, nearly 14,000 patients, and 261 agents reported that combination therapies were associated with substantially higher ORRs compared with monotherapy (15.8% vs. 3.5%). Additionally, ORRs varied by class of agent and disease type (9).

In this analysis, we focused exclusively on immunotherapy trials, which present additional complexities related to tumor size assessment and the unique features of cancer immunobiology. In this scenario, the use of DCR is a reasonable outcome, as durable SD is frequently observed with newer immunotherapeutic agents, related to their cytostatic mechanism of action (10). For example, in a phase II study of tebentafusp in patients with previously treated metastatic uveal melanoma, the agent demonstrated an ORR of only 5% yet still received FDA approval, largely due to its impact on OS (11). Especially for patients with colorectal cancer, we found an association between treatment with PPIs and reduced survival. This is consistent with prior retrospective (12) and metagenomic (13) investigations showcasing that PPIs can modify the composition of the gut microbiome (14), potentially through downstream translocation of oral commensals, thereby altering its immunomodulatory properties and impairing the efficacy of ICIs among patients with non–small cell lung cancer and other epithelial tumors (15, 16), particularly when used in monotherapy (17). Also, FPs targeting lipopolysaccharide, which is one of the most prevalent products in the gut microbiome and is associated with low rates of response to anti–PD-L1 ICIs, significantly improved the efficacy of anti–PD-L1 immunotherapy in patients with colorectal cancer (18).

Evidence of the deleterious effect of acetaminophen use on ICI outcomes for patients with solid tumors in the literature is robust (3, 19). In our analysis, only a limited number of patients received this agent as monotherapy; it was more commonly administered in combination with narcotics. As a result, the assessment of its independent impact on survival was constrained. Also, an important limitation of our study is that we were unable to ascertain the specific rationale underlying certain drug prescriptions, specifically whether the prescription was driven by treatment-related adverse events or other clinical considerations. We acknowledge the apparent discrepancy between the significant association of antihistamine use with improved OS and the lack of a corresponding benefit in PFS. This finding likely reflects several limitations inherent to the retrospective nature of our analysis. The study cohort was heterogeneous, and antihistamine use was not protocol-driven—varying in indication, timing, and dosage—which may have attenuated any measurable impact on early disease dynamics, as captured by PFS. Moreover, OS encompasses a broader range of influences beyond initial disease progression, including subsequent treatments and supportive care, which may disproportionately affect survival outcomes and potentially amplify marginal associations. As such, the observed OS signal should be interpreted cautiously. We believe these findings are exclusively hypothesis-generating and underscore the need for prospective validation in more controlled clinical settings. Furthermore, 10% of patients lacked evaluable response data, primarily owing to consent withdrawal or death prior to their initial response assessment.

In summary, the simultaneous use of antihistamines is associated with improved survival in patients undergoing experimental cancer immunotherapy. On the other hand, the use of PPIs within 30 days of the administration of an investigational immunotherapy agent is linked to reduced survival in individuals with colorectal cancer. These results underscore the influence of drug interactions on treatment response and survival, providing valuable insights for maximizing experimental immunotherapy efficacy.

## Supplementary Material

Supplementary Table S1 Supplementary Table S1. Drugs evaluated

## References

[1] Korman AJ , Garrett-Thomson SC, Lonberg N. The foundations of immune checkpoint blockade and the ipilimumab approval decennial. Nat Rev Drug Discov 2022;21:509–28. 34937915 10.1038/s41573-021-00345-8

[2] Zhang Y , Zhang Z. The history and advances in cancer immunotherapy: understanding the characteristics of tumor-infiltrating immune cells and their therapeutic implications. Cell Mol Immunol 2020;17:807–21. 32612154 10.1038/s41423-020-0488-6 PMC7395159

[3] Bessede A , Marabelle A, Guégan JP, Danlos FX, Cousin S, Peyraud F, . Impact of acetaminophen on the efficacy of immunotherapy in cancer patients. Ann Oncol 2022;33:909–15. 35654248 10.1016/j.annonc.2022.05.010

[4] Stefani A , Bria E. Is immunotherapy with concomitant proton pump inhibitor use a viable combination? JAMA Netw Open 2023;6:e2322922. 37432691 10.1001/jamanetworkopen.2023.22922

[5] Li H , Xiao Y, Li Q, Yao J, Yuan X, Zhang Y, . The allergy mediator histamine confers resistance to immunotherapy in cancer patients via activation of the macrophage histamine receptor H1. Cancer Cell 2022;40:36–52.e9. 34822775 10.1016/j.ccell.2021.11.002 PMC8779329

[6] Harvey RD , Mileham KF, Bhatnagar V, Brewer JR, Rahman A, Moravek C, . Modernizing clinical trial eligibility criteria: recommendations of the ASCO-Friends of Cancer Research washout period and concomitant medication work group. Clin Cancer Res 2021;27:2400–7. 33563635 10.1158/1078-0432.CCR-20-3855 PMC8102304

[7] Eisenhauer EA , Therasse P, Bogaerts J, Schwartz LH, Sargent D, Ford R, . New response evaluation criteria in solid tumours: revised RECIST guideline (version 1.1). Eur J Cancer 2009;45:228–47. 19097774 10.1016/j.ejca.2008.10.026

[8] Bader JE , Wolf MM, Lupica-Tondo GL, Madden MZ, Reinfeld BI, Arner EN, . Obesity induces PD-1 on macrophages to suppress anti-tumour immunity. Nature 2024;630:968–75. 38867043 10.1038/s41586-024-07529-3 PMC11456854

[9] Chihara D , Lin R, Flowers CR, Finnigan SR, Cordes LM, Fukuda Y, . Early drug development in solid tumours: analysis of National Cancer Institute-sponsored phase 1 trials. Lancet 2022;400:512–21. 35964611 10.1016/S0140-6736(22)01390-3 PMC9477645

[10] Gouda MA , Ballesteros PA, Garrido-Laguna I, Rodon J. Efficacy assessment in phase I clinical trials: endpoints and challenges. Ann Oncol 2025;36:507–19. 40049448 10.1016/j.annonc.2025.02.010 PMC13634258

[11] Carvajal RD , Butler MO, Shoushtari AN, Hassel JC, Ikeguchi A, Hernandez-Aya L, . Clinical and molecular response to tebentafusp in previously treated patients with metastatic uveal melanoma: a phase 2 trial. Nat Med 2022;28:2364–73. 36229663 10.1038/s41591-022-02015-7 PMC9671803

[12] Lopes S , Pabst L, Dory A, Klotz M, Gourieux B, Michel B, . Do proton pump inhibitors alter the response to immune checkpoint inhibitors in cancer patients? A meta-analysis. Front Immunol 2023;14:1070076. 36776847 10.3389/fimmu.2023.1070076 PMC9910608

[13] Derosa L , Routy B, Thomas AM, Iebba V, Zalcman G, Friard S, . Intestinal Akkermansia muciniphila predicts clinical response to PD-1 blockade in patients with advanced non-small-cell lung cancer. Nat Med 2022;28:315–24. 35115705 10.1038/s41591-021-01655-5 PMC9330544

[14] Imhann F , Bonder MJ, Vila AV, Fu J, Mujagic Z, Vork L, . Proton pump inhibitors affect the gut microbiome. Gut 2016;65:740–8. 26657899 10.1136/gutjnl-2015-310376 PMC4853569

[15] Routy B , Le Chatelier E, Derosa L, Duong CPM, Alou MT, Daillère R, . Gut microbiome influences efficacy of PD-1–based immunotherapy against epithelial tumors. Science 2018;359:91–7. 29097494 10.1126/science.aan3706

[16] Zhou C-B , Zhou Y-L, Fang J-Y. Gut microbiota in cancer immune response and immunotherapy. Trends Cancer 2021;7:647–60. 33674230 10.1016/j.trecan.2021.01.010

[17] Kawachi H , Yamada T, Tamiya M, Negi Y, Goto Y, Nakao A, . Concomitant proton pump inhibitor use with pembrolizumab monotherapy vs immune checkpoint inhibitor plus chemotherapy in patients with Non−Small cell lung cancer. JAMA Netw Open 2023;6:e2322915. 37432682 10.1001/jamanetworkopen.2023.22915 PMC10336622

[18] Song W , Tiruthani K, Wang Y, Shen L, Hu M, Dorosheva O, . Trapping of lipopolysaccharide to promote immunotherapy against colorectal cancer and attenuate liver metastasis. Adv Mater 2018;30:1805007. 10.1002/adma.201805007 PMC6580426 30387230

[19] Nelli F , Virtuoso A, Giannarelli D, Fabbri A, Giron Berrios JR, Marrucci E, . Effects of acetaminophen exposure on outcomes of patients receiving immune checkpoint inhibitors for advanced non-small-cell lung cancer: a propensity score-matched analysis. Curr Oncol 2023;30:8117–33. 37754504 10.3390/curroncol30090589 PMC10527930

