# Supplementary material for: Modulating Treatment Outcomes of Patients with Solid Tumors in Immunotherapy Trials: A Drug Interaction Analysis from a Phase I Unit
Source: Cancer Res Commun. 2025 Sep 15;5(9):1631–41. doi: 10.1158/2767-9764.CRC-25-0033 (PMC12434680; doi:10.1158/2767-9764.CRC-25-0033)
Supplement: Supplementary Table S1 — Drugs evaluated [file crc-25-0033_supplementary_table_s1_suppst1.docx]

**Supplementary Table S1 – Drugs evaluated**

| **Class** | **Generic name** | **Possible brand names** |
| --- | --- | --- |
| NSAIDs | Acetaminophen | Tylenol |
|  | Aspirin | Too many brand names, generic search |
|  | Celecoxib | Celebrex |
|  | Diclofenac | Cambia, Lofena, Zipsor |
|  | Diflunisal | Generic only |
|  | Etodolac | Lodine |
|  | Fenoprofen | Nalfon |
|  | Ibuprofen | Many brand names (most common: Motrin, Advil) |
|  | Indomethacin | Indocin |
|  | Ketoprofen | Kiprofen |
|  | Ketorolac | Toradol |
|  | Meclofenamate | Generic only |
|  | Meloxicam | Mobic |
|  | Nabumetone | Relafen |
|  | Naproxen | Many brand names (most common: Aleve, Naprosyn) |
|  | Oxaprozin | Daypro |
|  | Piroxicam | Feldene |
|  | Sulindac | Clinoril |
|  | Tolmetin | Tolectin |
| Antihistamines | Carbinoxamine | Karbinal, RyVent |
|  | Cetirizine | Zyrtec |
|  | Chlorpheniramine | Chlor-Trimeton |
|  | Clemastine | Generic only |
|  | Cyproheptadine | Periactin |
|  | Desloratadine | Clarinex |
|  | Dexchlorpheniramine | RyClora |
|  | Dimenhydrinate | Dramamine |
|  | Diphenhydramine | Many brand names (most common: Benadryl, Banophen) |
|  | Doxylamine | Unisom |
|  | Fexofenadine | Allegra |
|  | Hydroxyzine | Atarax, Vistaril |
|  | Levocetirizine | Xyzal |
|  | Loratadine | Claritin |
|  | Meclizine | Antivert, Bonine |
|  | Triprolidine | Histex |
| Steroids | Budesonide | Entocort, Tarpeyo, Uceris |
|  | Cortisone | Generic only |
|  | Deflazacort | Emflaza |
|  | Dexamethasone | Decadron |
|  | Fludrocortisone | Florinef |
|  | Hydrocortisone | Cortef, Solu-Cortef |
|  | Methylprednisolone | Medrol dosepak, Solu-Medrol |
|  | Prednisolone | Orapred |
|  | Prednisone | Rayos |
|  | Vamorolone | Agamree |
| PPIs | Dexlansoprazole | Dexilant |
|  | Esomeprazole | Nexium |
|  | Lansoprazole | Prevacid |
|  | Omeprazole | Prilosec |
|  | Pantoprazole | Protonix |
|  | Rabeprazole | Aciphex |
| Antibiotics | Amoxicillin | Amoxil |
|  | Amoxicillin/clavulanate | Augmentin |
|  | Ampicillin | Generic only |
|  | Ampicillin/sulbactam | Unasyn |
|  | Atovaquone | Mepron |
|  | Azithromycin | Z-pak, Zithromax |
|  | Cefdinir | Omnicef |
|  | Cefepime | Maxime |
|  | Cefpodoxime | Vantin |
|  | Ceftriaxone | Rocephin |
|  | Cefuroxime | Ceftin |
|  | Cephalexin | Keflex |
|  | Ciprofloxacin | Cipro |
|  | Doxycycline | Doryx, Doxy |
|  | Levofloxacin | Levaquin |
|  | Metronidazole | Flagyl |
|  | Minocycline | Minocin |
|  | Nitrofurantoin | Macrobid |
|  | Penicillin | Pen-VK, Veetids |
|  | Pentamidine | Pentam |
|  | Piperacillin/tazobactam | Zosyn |
|  | Sulfamethoxazole/trimethoprim | Bactrim, Bactrim DS |
|  | Tetracycline | Generic only |
| Antidepressants | Amitriptyline | Elavil |
|  | Buproprion | Wellbutrin |
|  | Citalopram | Celexa |
|  | Desvenlafaxine | Pristiq |
|  | Duloxetine | Cymbalta |
|  | Escitalopram | Lexapro |
|  | Fluoxetine | Prozac |
|  | Nortriptyline | Pamelor |
|  | Paroxetine | Paxil |
|  | Sertraline | Zoloft |
|  | Trazodone | Desyrel |
|  | Venlafaxine | Effexor |
|  | Vilazodone | Viibryd |
|  | Vortioxetine | Trintellix |
| Antiemetics | Aprepitant | Emend |
|  | Dronabinol | Marinol, Syndros |
|  | Fosaprepitant | Emend, Focinvez |
|  | Granisetron | Kytril, Sancuso |
|  | Lorazepam | Ativan |
|  | Metoclopramide | Reglan |
|  | Nabilone | Cesamet |
|  | Netupitant and palonosetron | Akynzeo |
|  | Olanzapine | Zyprexa |
|  | Ondansetron | Zofran |
|  | Palonosetron | Aloxi |
|  | Prochlorperazine | Compazine |
|  | Promethazine | Phenergan |
|  | Scopolamine | Transderm-Scop |
| Antidiabetics | Canagliflozin | Invokana |
|  | Dapagliflozin | Farxiga |
|  | Dulaglutide | Trulicity |
|  | Empagliflozin | Jardiance |
|  | Exenatide | Byetta |
|  | Glimepiride | Amaryl |
|  | Glipizide | Glucotrol |
|  | Glyburide | Glynase |
|  | Insulin | Too many brand names, generic search |
|  | Linagliptin | Tradjenta |
|  | Liraglutide | Victoza |
|  | Lixisenatide | Adlyxin |
|  | Metformin | Glucophage |
|  | Nateglinide | Starlix |
|  | Pioglitazone | Actos |
|  | Rosiglitazone | Avandia |
|  | Saxagliptin | Onglyza |
|  | Semaglutide | Ozempic, Rybelsus, Wegovy |
|  | Sitagliptin | Januvia |
| Anticoagulants | Apixaban | Eliquis |
|  | Dabigatran | Pradaxa |
|  | Dalteparin | Fragmin |
|  | Edoxaban | Savaysa |
|  | Enoxaparin | Lovenox |
|  | Rivaroxaban | Xarelto |
| Immunosuppressants | Infliximab | Inflectra, Remicade |
|  | Rituximab | Rituxan (not for cancer treatment) |
|  | Vedolizumab | Entyvio |
|  | Tocilizumab | Actemra |
| Bone-modifying agents | Denosumab | Xgeva |
|  | Zoledronic acid | Zometa |
| Narcotics | Fentanyl | Duragesic, Actiq, Fentora |
|  | Meperidine | Demerol |
|  | Oxycodone | OxyIR, Oxycontin long-acting, Xtampza ER long-acting |
|  | Morphine | MSIR, MS Contin long-acting, Kadian long-acting |
|  | Hydromorphone | Dilaudid, Exalgo long-acting |
|  | Oxymorphone | No brand name currently on U.S. market |
|  | Hydrocodone + acetaminophen | Norco, Lortab, Vicodin |
|  | Methadone | Dolophine |
|  | Tramadol | Ultram, Qdolo |
|  | Codeine + acetaminophen | Tylenol |
